# Stem cell-derived intestinal organoids: a novel modality for IBD

**DOI:** 10.1038/s41420-023-01556-1

**Published:** 2023-07-21

**Authors:** Cheng-mei Tian, Mei-feng Yang, Hao-ming Xu, Min-zheng Zhu, Ning-Ning Yue, Yuan Zhang, Rui-yue Shi, Jun Yao, Li-sheng Wang, Yu-jie Liang, De-feng Li

**Affiliations:** 1 Department of Gastroenterology, Shenzhen People’s Hospital, The Second Clinical Medical College, Jinan University; The First Affiliated Hospital, Southern University of Science and Technology, Shenzhen, 518020 Guangdong China; 2 Department of Emergency, Shenzhen People’s Hospital, The Second Clinical Medical College, Jinan University; The First Affiliated Hospital, Southern University of Science and Technology, Shenzhen, 518020 Guangdong China; 3 Department of Hematology, Yantian District People’s Hospital, Shenzhen, 518020 Guangdong China; 4 Department of Gastroenterology and Hepatology, Guangzhou Digestive Disease Center, Guangzhou First People’s Hospital, School of Medicine, South China University of Technology, Guangzhou, 51000 China; 5 Department of Gastroenterology, Shenzhen People’s Hospital The Second Clinical Medical College, Jinan University, Shenzhen, 518020 Guangdong China; 6 Department of Medical Administration, Huizhou Institute of Occupational Diseases Control and Prevention, Huizhou, 516000 Guangdong China; 7 Department of Child and Adolescent Psychiatry, Shenzhen Kangning Hospital, Shenzhen, 518020 Guangdong China

**Keywords:** Haematopoietic stem cells, Inflammatory bowel disease

## Abstract

The organoids represent one of the greatest revolutions in the biomedical field in the past decade. This three-dimensional (3D) micro-organ cultured in vitro has a structure highly similar to that of the tissue and organ. Using the regeneration ability of stem cells, a 3D organ-like structure called intestinal organoids is established, which can mimic the characteristics of real intestinal organs, including morphology, function, and personalized response to specific stimuli. Here, we discuss current stem cell-based organ-like 3D intestinal models, including understanding the molecular pathophysiology, high-throughput screening drugs, drug efficacy testing, toxicological evaluation, and organ-based regeneration of inflammatory bowel disease (IBD). We summarize the advances and limitations of the state-of-the-art reconstruction platforms for intestinal organoids. The challenges, advantages, and prospects of intestinal organs as an in vitro model system for precision medicine are also discussed.

**Figure:**
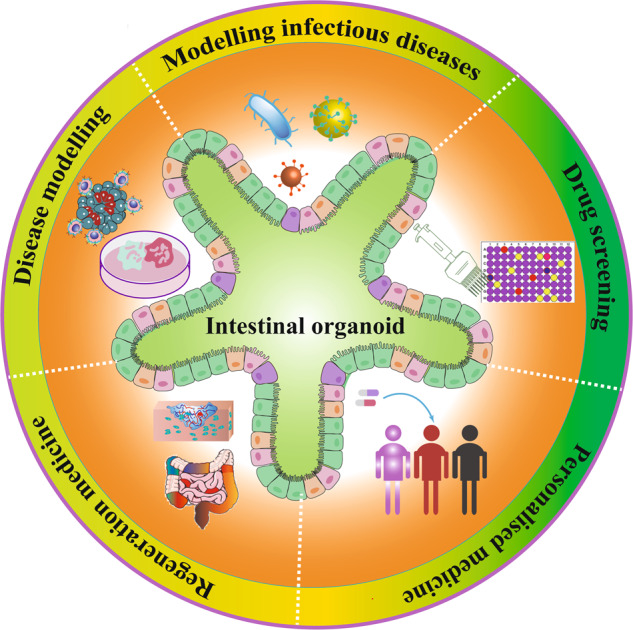
Key applications of stem cell-derived intestinal organoids. Intestinal organoids can be used to model infectious diseases, develop new treatments, drug screens, precision medicine, and regenerative medicine.

Key applications of stem cell-derived intestinal organoids. Intestinal organoids can be used to model infectious diseases, develop new treatments, drug screens, precision medicine, and regenerative medicine.

## Facts

- ISCs in the intestinal crypts maintain the integrity of the intestinal epithelium.
- ISCs or PSCs can self-organize and differentiate into “intestinal organoids” by specific 3D culture.
- SCs-derived intestinal organoids are ideal models of the intestinal tract and have obvious advantages in the study of intestinal physiology and intestinal diseases.
- SCs-derived intestinal organoids have been successfully transplanted into the intestinal mucosa and have been shown to have a repairing effect on the intestinal epithelium, which holds great prospects for the treatment of intestinal diseases.

## Open questions

- How to optimize the culture system and culture process of intestinal organoids?
- How to improve the simulation and homogeneity of intestinal organoid models?
- Can new therapeutic targets be identified using intestinal organoids?
- Can intestinal organoid transplantation be a method to repair intestinal epithelial damage?

## Introduction

**Fig. 1:**
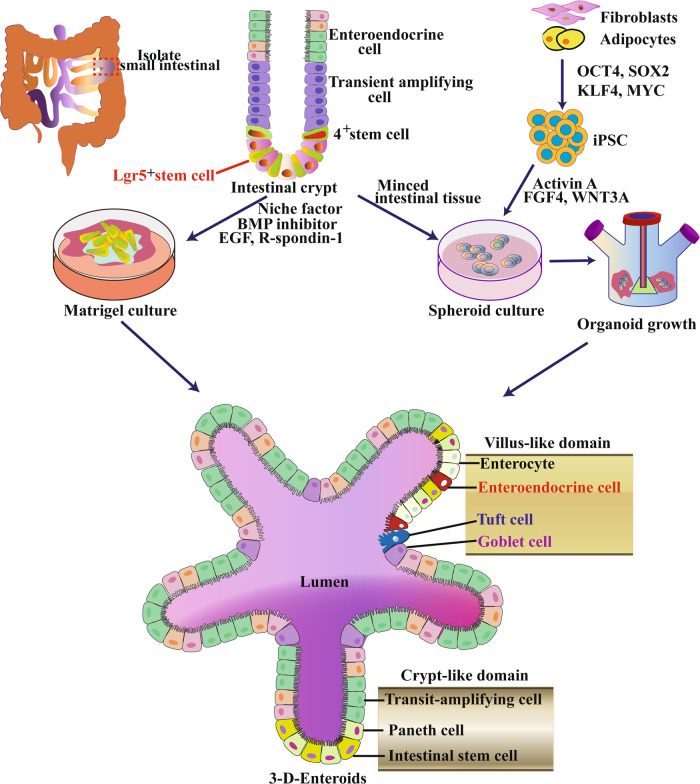
Schematic diagram of intestinal tissue engineering by organoid technology. Intestinal organoid is generated from the intestinal crypts of the small intestine, and Lgr5+ stem cells were isolated from small intestinal tissues are then embedded in Matrigel with a culture medium. Clonal fibroblast subclones and clonal iPSCs were established in the parental fibroblast population and then co-generated with fragmented intestinal tissue on 3D scaffold material to generate intestinal organoids.

Inflammatory bowel disease (IBD) is a chronic disease with an increasing incidence worldwide [1]. The intestinal epithelial barrier plays a major role in IBD [2, 3]. However, the lack of techniques for long-term culture of human primary epithelial cells in vitro hinders the study of the role of intestinal epithelium in IBD. In recent years, intestinal stem cells (ISCs) have become the focus of research on intestinal injury and regeneration, colorectal cancer, and other intestinal diseases [4]. At present, leucine rich repeat containing G protein coupled receptor 5+ (Lgr5+) stem cells (SCs) play an important role in maintaining normal intestinal epithelial structure by renewing the intestinal epithelium by generating billions of cells at an alarming rate every day [5]. Studies have shown that ISCs and crypts in vitro can form hollow spheres with intact intestinal epithelial-like structures, known as “intestinal organoids”, through 3D culture mode in matrix glue (Matrigel) under the action of appropriate growth factors [6]. In addition, pluripotent SCs (PSC) or induced pluripotent SCs (iPSC) derived from patients with IBD are also capable of forming intestinal organoids under in vitro induction. These organs are hollow globules with complete intestinal epithelioid structures. Intestinal epithelial organs cultured in vitro for several months retain their cellular characteristics and functions because they are fully integrated into the recipient epithelial cells [7–9]. They retain their original cell characteristics and functions even after several months of in vitro culture, making them ideal physiological models of the intestinal epithelium and unique disease models, and can be transplanted intact into the recipient intestinal mucosa to achieve repair of intestinal epithelial damage [10, 11]. Cellular sources and methods for the generation of intestinal organoid have illustrated in Fig. 1.

This review discusses the progress of research on the formation of intestinal organoids by SCs in culture. And discusses the research progress and current limitations of intestinal organoids in establishing intestinal epithelial models and treating patients with IBD. Finally, we outline the urgent need to technically standardize laboratory procedures for intestinal organs in order to make them more widely used in clinical IBD studies.

## IBD intestinal model

Clinical studies and applications of IBD are subject to strict technical and ethical restrictions, and high-quality preclinical studies are needed to ensure the efficacy and safety of clinical studies [12, 13]. The main preclinical research models for IBD are animal models and cellular models. For a long time, animal models have been the mainstay of intestinal research due to the lack of representativeness of cellular models [14]. However, animal models have long time cycles and racial differences from humans with being expensive and subject to animal ethics [14]. Therefore, it requires effective, safe, and feasible cellular models to avoid unnecessary animal experiments as much as possible. Besides, animal models are difficult to be widely carried out in disease research [15, 16]. The intestinal cell model is limited by the technology of intestinal epithelial cell culture, and the mechanism of intestinal epithelial damage for IBD patients is under-researched and the treatment methods are limited. In the past decade or so, SCs-derived intestinal organoid models have brought a leap forward in IBD research and can provide new directions and methods for IBD mechanism research and treatment development [17, 18].

### 2D cellular model

The in vitro cell model is to isolate primary cells from in vivo tissues, perform cell culture and generate different types of cell lines [19, 20]. Until the ISCs were isolated, the intestinal cell model could only be replaced by intestinal tumor cells. The developed Caco-2, HT-29, and T84 cell lines were derived from human colorectal adenocarcinoma [21–24].

The 2D cell culture environment is far from the complex cellular environment in vivo, which affects the structure and function of cells [21]. 2D culture cell lines have few cell types and confusing tissue structure, and the cell lines will gradually flatten, abnormally differentiate and lose their differentiation phenotype, which cannot reflect the interaction between different cells and matrix, and cannot form similar cellular tissue structure in vivo. It cannot reflect the interactions between different cells and matrix, and cannot form similar cellular tissue structures in vivo [20, 25]. It is generally accepted that 2D cell models are difficult to reflect the in vivo situation, have little reference value, and the experimental results are not widely accepted [26–28].

### 3D cellular model

3D cell culture is an in vitro co-culture of carriers with 3D structures and different materials with different cell types so that the cells can migrate and grow in the carriers with 3D spatial structure, forming 3D cell-carrier complexes [28, 29]. A 3D cell model optimizes the limitations of a 2D cell model. 3D culture environment can better simulate the in vivo cell growth environment, mimic the in vivo cell growth environment, regulate cell proliferation and differentiation, and cultivate more cell types to form tissue structures similar to in vivo [30]. Currently, 3D cell models are widely used in various fields such as organoid, microtumor, and microcarrier [31, 32].

Lgr5(+) ISCs were successfully isolated in 2007, and have become the most important cell source for intestinal cell models [33]. In vivo, Lgr5+ crypt base columnar cells (CBC) are located at the base of the intestinal mucosal crypt and differentiate to form different intestinal mucosal cells by continuously proliferating and migrating toward the top of the crypt [34]. In vitro, Lgr5(+) CBC has a short survival time and cannot be cultured in a 2D culture environment, but can survive for a long time in a 3D culture environment [35]. In 3D culture, Lgr5+ CBC was found to divide once every 24 h and could differentiate into all intestinal epithelial cells (IEC) except mesenchymal cells and immune cells [36].

### Intestinal organoid model

**Fig. 2:**
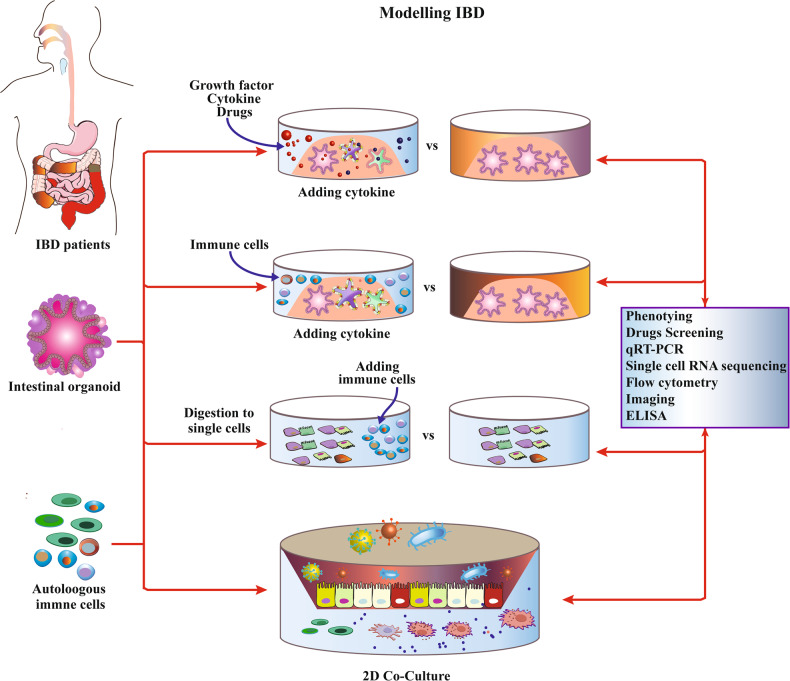
Intestinal Organoids as models for IBD research. Intestinal organoids can be used for intestinal development and IBD disease modeling, drug/toxicity testing, and host-pathogen interaction studies. In cultures of intestinal organoids from IBD patients, we can add multiple growth factors, cytokines, or drug molecules to modulate the culture environment, add autoimmune cells for co-culture, or digest into single cells. The functions can evaluate by phenotypic analysis, drug screening, qRT-PCR, single cell RNA sequencing, flow cytometry, imaging, ELISA, and other kinds of indicators.

Intestinal organoid is a miniature hollow sphere with an intestinal epithelial crypt structure formed by SCs in 3D culture, which mimics the ecological niche of ISCs, and precisely regulates the proliferation and differentiation of SCs [17, 37–48]. These spheres contain most types of IEC, and when differentiated mature, they have physiological functions such as absorption, secretion, mucus production, and material transport [49, 50]. Intestinal organoids have short culture cycles, can be stored frozen for long periods of time, and can also simulate complex environments in vivo or be adapted to the culture environment. Intestinal organoids are able to preserve the intestinal epithelial crypt structure and maintain stable phenotypic and genetic characteristics [7, 43, 51–53]. Compared with cell line models, organoids have similar tissue structure and function to those in vivo, improving the realism and reliability of the study [18, 27]. In addition, the culture cycle is shorter than that of animal models, the process is easy to manipulate, and there are no animal ethical issues involved, allowing for a wider range of studies [16, 29, 54, 55]. Table 1 Summary of current intestine models. Figure 2 Intestinal Organoids as models for IBD research.

**Table 1:** Summary of current intestine models.

Intestinal models	Advantage	Limitation	Ref.
Intestine animal models	Traditional model; Technology maturity; Approaching the stage of clinical studies.	long time cycles; expensive; racial differences from human; subject to animal ethics.	[15, 16]
2D intestine cellular model	The culture is simple, the technology is mature, the cost is low, and it is adapted to simple preclinical studies.	Planar culture environment, monolayer cell culture, gradually planarizing and losing differentiation phenotype, inability to form intestinal epithelial structures.	[168–173]
3D intestine organoid model	3D culture environment that mimics the growth environment of ISCs in vivo, forms intestinal epithelial structures, and maintains stable phenotypic and genetic characteristics.	Culture process and technology is relatively complex, and cost is relatively high. There are still limitations that hinder clinical translation.	[174–178]
Intestine Organoid microarray	More precise regulation of the cultivation environment, simulating the physical and microbial environment in the body.	The culture system and process lack standards, the culture technology needs to be improved, and the cost is high.	[179–184]

The cell sources of intestinal organoids include ISCs from intestinal crypts, PSCs, embryonic SCs (ESCs), and iPSCs [18, 56]. In 2009, Hans et al. reported for the first time in Nature the formation of murine intestinal organoids with intestinal epithelial crypt structure using Lgr5(+) ISCs from mouse intestinal crypt in vitro [6]. In 2011, Hans et al. cultured human intestinal organoids with intestinal epithelial crypt structure using Lgr5(+) ISCs from human intestinal crypt [47]. After more than a decade of development, ISCs-derived intestinal organoid cultures have matured with high success rates, and are currently the main source of intestinal organoid cultures [57, 58]. However, the cultures do not contain MSCs and immune cells [42, 59]. In 2011, Spence et al. reported the use of PSCs or embryonic SCs to culture human intestinal organoids [10]. In 2017, Miura and Suzuki reported the formation of intestinal organoids using mouse and human iPSCs cultures, respectively [60]. PSC, ESC, and iPSC cultures form intestinal organoids with crypt structures of the intestinal epithelium and contain various types of IEC, but the differentiated IEC is not sufficiently mature [61, 62]. There is a potential risk of genetic and epigenetic variation during induction, with some differences in structure, function, and genetic characteristics from the IEC in vivo [60, 62]. Currently, the success rate of various PSCs cultured to form intestinal organoids is low and the technique is still flawed, pending subsequent improvements [10, 43, 61].

## Intestinal organoid culture technology

Organoid culture technology was named one of the "Top Ten Breakthroughs" by Science in 2013 and Nature Methods in 2017, and an excellent preclinical disease model by The New England Journal of Medicine in 2019 [63]. The organoid culture technology includes core technologies such as 3D culture, detection, and identification, as well as basic technologies such as stem cell isolation, organoid extraction, and organoid preservation [58, 64]. In addition, emerging technologies such as 3D bioprinting, organoid microarrays, and gene editing technologies can be combined to increase environmental simulation conditions or expand the scope of research [53, 65, 66]. To better simulate the intestinal epithelial growth environment in vivo, organoid culture is systematized and a 3D culture system for intestinal organoids is established [47, 67–69]. The current 3D organoid culture system can be divided into scaffold-free culture system and scaffold-based culture system according to the presence or absence of scaffold deriving from natural components or artificially synthesized [70].

### scaffold-free culture system

The scaffold-free culture system has no support structures for cell adhesion, growth, and spreading [64]. The 3D culture environment is formed by various physicochemical principles, and the cells aggregate in the medium to form microtissue spheroids similar to the source tissue [55, 64]. Hanging drops, which have no plane of attachment, can be prepared as a variety of hanging drop plates allowing cells to self-assemble under the influence of gravity to form microtissue spheroids [71–73]. The cells can be magnetized and suspended in a magnetic field for 3D culture [74–76]. Special synthetic polymer materials can be used to form microplate structures with ultra-low adhesion surfaces on which the cells migrate and adhere to each other to form microtissue spheroids [64, 77]. Alternatively, the agar interface can be used to reduce the stiffness of the culture surface to create a 3D Petri Dish in which cells can migrate and spread to form microtissue spheroids [43, 78].

### Scaffold-based culture system

Scaffold-based culture systems allow cells to attach to a scaffold composed of solid particles or liquid gels, which are made to float in the culture medium by gentle agitation [38]. With the development of technology, scaffold appears in more and more forms to solve different problems [67]. According to the source of scaffold, it can be divided into scaffold supported by natural extracellular matrix (ECM) and scaffold supported by synthetic materials [38, 67, 79].

#### Natural ECM-supported scaffold

Natural ECM is used as the support material to optimize the 3D culture matrix formulation according to the cultured cell types meeting the culture needs of different tissue cells [80]. Matrix gel is a natural ECM extracted from the basement membrane of mouse sarcoma cells in liquid gel form, which is a natural scaffold with good compatibility for both human and mouse cell cultures [80, 81]. The main components of matrix gel are laminin, type IV collagen, nestin, heparan sulfate glycoprotein, and also contains growth factors and matrix metalloproteinases, providing rich nutrition [43]. However, natural ECM has some immunogenicity and risk of pathogenic infection and is less stable [82]. Natural ECM may have the disadvantage of batch variability due to different matrices and extraction techniques [81, 83].

#### Synthesized material-supported scaffold

The types of synthetic scaffold materials are quite diverse. Synthetic materials have consistency, stability, and biocompatibility bias, and cannot provide growth factors and small molecule compounds needed for cell culture. The synthetic scaffold can be divided into hydrogel and solid scaffold according to the attachment method of cells [67, 79].

##### Hydrogels

The hydrogel is mimics of ECM and can be made from natural polymers or synthetic polymers [64]. SCs are dispersed in liquid hydrogels and then cross-linked to achieve 3D culture [38, 84]. The hydrogel scaffold has good stability and adjustability [29, 38, 64]. Hydrogels can alter cell signaling by increasing or decreasing protein concentration and changing the density of cell adhesion ligands [39, 66]. Polymeric gelling agents can be pre-designed, and functional additives can be adjusted as required, allowing the hydrogel to obtain specific properties such as temperature, magnetic properties, and pH, and meet a wider range of research requirements [39, 40, 67].

##### Solid scaffold

Solid scaffold uses a variety of porous materials to prepare a solid, porous microcarrier structure [85, 86]. SCs are "seeded" in these solid scaffold porous microcarriers to achieve a 3D culture environment [86, 87]. Sponges or foams have high porosity and homogeneous connectivity structures, and the prepared porous microcarrier scaffolds are widely used in tissue engineering [85, 88]. Solid scaffolds prepared by applying biodegradable polymers, such as PLA, have been extensively studied for their good biocompatibility and proper porous structure [89, 90]. Enables efficient 3D cell attachment for superior cell viability [67, 89, 91].

### Organoid detection and identification techniques

Intestinal organoids are microtissue spheroids with intestinal epithelial structure, and their detection and identification techniques are different from traditional 2D cell lines [58]. It is necessary to evaluate the quality of intestinal spheroids in culture, as well as to evaluate the research results of intestinal spheroid models. The development of various characterization techniques and assays has enriched the identification and evaluation of organoids. The application and promotion of intestinal organoid technology in the study of intestinal diseases have been promoted [29, 47, 58, 92]. The tests that can be performed now include morphological structure, proliferative activity, intestinal epithelial barrier function, and genetics [93–95].

### Organoid microarray technology

Stationary 3D cell culture is performed in culture dishes or culture plates, and the culture environment that can be regulated is very limited [96]. The intestinal epithelial cells are regulated by multiple systems of the organism and are also influenced by the intestinal microecology and microenvironment [97, 98]. Organoid microarrays are microfluidic microarray technology combined with 3D organoid culture technology to form a dynamic 3D cell culture environment in vitro by more rigorously mimicking the functional units of human tissues and organs in a miniature organoid culture vehicle [41, 99]. The organoid chip can simulate the physical environment such as in vivo mechanics, and magnetic and electric fields by dynamically regulating the culture medium and additives through microfluidics [100]. It can add different cells or microorganisms to mediate cell-cell and cell-microbe interactions [100–102]. Microarray technology can also be combined with imaging instruments to monitor cell biological changes in real time, record behavioral changes of cells in disease states, and record the whole process of cellular response to drugs [103–105]. Microarray technology enables more systematic and mechanized organoid culture, increases throughput, expands the scope of research, and takes a firm step toward clinical translation [69, 100, 106–108].

### Bio-3D printing technology

Bio-3D printing technology has broken through cell printing and manipulated cell-containing bio-ink to construct active structures [109, 110]. The hybrid printing of SCs with biological scaffolds through multi-jet 3D printing technology to form organoid structures could be the next generation of organoid construction methods [111]. It has been shown that ‘assembloids’ assembled by cell-based 3D printing technology go beyond Organoid and are closer to human tissues and organs in terms of structure and function [112]. At present, bio-3D printing is mainly used for in vitro research models, for forming 3D culture systems, organoid chips, and printing biomimetic materials, such as gelatin, alginate, and creating conditions for in vitro culture of SCs into desirable organoids [113]. Currently, although it is possible to print cell-containing structures that resemble in vivo tissues and organs in shape and structure, there is still a big gap between them and the complex physiological functions of real organs. There is still quite a long way to go before active organs can be printed using for transplantation [68, 77, 114].

## Intestinal organoid applications

The applications of Intestinal organoids are focused on both research models and clinical treatments. The intestinal organoid has the structural characteristics and physiological functions of the intestinal epithelium, which is an ideal model of intestinal epithelial physiology and intestinal diseases [55]. The organoid is able to maintain stable phenotypic and genetic characteristics, and has obvious advantages in individualized disease modeling and precision medicine [7, 115]. As an intestinal epithelial physiological model, the growth and development, tissue structure, and physiological function of intestinal epithelium can be studied. As a model of IBD disease, it can explore the pathogenesis of IBD and test the treatment effect of IBD. Intestinal organoids can be used in IBD treatment to repair intestinal epithelial damage through organoid transplantation and to improve genetic susceptibility and gene expression through gene therapy [17, 50, 53, 116].

### Studying intestinal physiology

Intestinal organoids maintain the structure and function of the intestinal epithelium [29]. By observing the formation process of the organoid, the complex environmental changes in vivo are simulated, thus understanding the structure and physiological functions of the intestinal epithelium. It also allows in-depth study of the proteomics, lipidomics, genomics, and transcriptomics of intestinal epithelial cells [29, 36, 63, 117, 118].

It was found that mature absorptive cells, cupped cells, and various intestinal endocrine cells could be detected in intestinal epithelial organoids derived from Lgr5(+) CBC [36, 117]. These cells have corresponding physiological functions such as transporting substances, absorbing nutrients, and synthesizing secretion [57, 59]. By comparing colonic epithelial organoids from healthy humans with those from patients with ulcerative colitis (UC), Dotti et al. found differences in DNA methylation profiles and gene expression profiles. suggesting that the UC colonic organoid model retains a disease-specific genetic and expression profile [7, 9, 115].

### Exploring the pathogenesis of IBD

Currently, the etiology and pathogenesis of IBD are unclear. In order to explore the pathogenesis of IBD and find the etiology of intestinal injury, colonic organoids are ideal models for disease research. Being able to establish an individualized disease model, preserving the morphological structure and genetic characteristics of diseased tissue cells, can lead to more realistic and reliable research results [9, 29].

#### In terms of genetics

A study comparing colonic epithelial organoids of terminal ileal origin from UC patients and Crohn’s disease (CD) patients found that they differ in DNA methylation profiles and gene expression profiles, although they have similar morphological structures [9]. It indicates that there are different disease-related genetic genes and expression profiles in each of UC and CD, which can provide a basis for subsequent differential diagnosis and gene therapy for IBD [7, 9, 93].

#### In terms of mucosal inflammatory damage

A study found that transcript levels of IL-1β and γ-IFN were lower in colonic organoid cultures than in their source mucosal biopsy specimens. The investigators concluded that the levels of inflammatory factors in mucosal specimens do not carry over to colonic organoids and that overexpression of these inflammatory factors requires re-stimulation [52]. In 2018, Biton et al. studied Th and its inflammatory factors in intestinal organoids on the proliferation and biochemistry of Lgr5(+) CBC [119]. Lgr5(+) CBC expressing major histocompatibility complex II (MHC II) was found to be an atypical antigen-presenting cell that interacts with Th via MHC II. Th1, Th2, Th17 and their secreted pro-inflammatory factors IFN-γ, IL-13 and IL-17A promote Lgr5(+) CBC differentiation [119, 120]. And regulatory Th (Treg) and the base-secreted cytokine IL-10 promote Lgr5(+) CBC regeneration [119]. A study found that the regenerative capacity of ISCs and the rate of organoid formation were reduced after short-term intervention of intestinal organoid Interleukin-22 (IL-22), but the number of transiently expanded progenitor cells increased and differentiated to various types of epithelial cells, resulting in an increase in the volume of formed organoids [121, 122]. It is hypothesized that IL-22 contributes to the differentiation of transiently expanded progenitor cells to various types of epitheliums, and promotes intestinal epithelial repair during acute injury of intestinal epithelium [121, 123]. However, IL-22 may be detrimental to epithelial regeneration and repair by causing damage to ISCs when chronic injury hampering the intestinal epithelium [123, 124]. The clinical application of IL-22 to promote mucosal repair in IBD patients should take into account its long-term chronic damage to ISCs [121].

#### In terms of intestinal flora

Wang et al. combined 3D differentiation technology at the air-liquid interface to construct an intestinal epithelial organoid and cell co-culture system to simulate pseudomembranous colitis caused by *C. difficile* infection of the intestine and to explore the pathogenic mechanism of *C. difficile* [125]. Hou et al. established a co-culture system of Lactobacillus, intestinal lamina propria lymphocytes, and intestinal organoid, and found Lactobacillus stimulated the proliferation and repair of intestinal epithelium of Intestinal organoids by promoting the secretion of IL-22 and TNF-α from lymphocytes in the lamina propria of the intestine [126].

#### In terms of fibrosis and cancerization

One study isolated the crypt from the mouse colon and cultured it into intestinal organoids. Tumor necrosis factor-α (TNF-α) stimulation and Transforming growth factor-β1 (TGF-β1) stimulation were successively administered to intestinal organoids to induce IEC mesenchymal cell transformation, providing a convenient and effective in vitro model for studying intestinal fibrosis in IBD [127]. In another study, cytokines (TNF-α, IL-1β, IL-6) and bacterial components (bacterial lipopolysaccharide, flagellin) were mixed and added to mouse colonic organoid medium on alternate days to simulate chronic inflammatory stimuli for more than 1 year of continuous intervention [128]. The results revealed that colonic organoid underwent a cellular transformation that may be associated with UC carcinogenesis after chronic inflammatory stimulation due to activation of the nuclear factor kappa-B (NF-κB) signaling pathway and impaired cell differentiation [128].

### Testing the effect of IBD treatment

IBD is mainly treated with drugs, and the application of colonic organoid models for drug testing is ideal. Colonic epithelial organoids can be cultured to simulate a more reviewed in vivo environment, and have a very high structural and functional similarity to the in vivo intestinal epithelium. It is highly realistic and reliable as a research model to study drug responses to intestinal epithelial structures. There is a significant increase in the success rate of clinical translation of IBD therapeutic agents or treatments that pass the test in the colonic organoid model [12, 118, 129, 130]. In precision medicine, colonic organoids derived from ISCs or iPSCs established from IBD patients are mostly used for drug testing [118]. Testing conventional therapeutic drugs and exploring new drugs for IBD treatment can help identify more optimal options for IBD patients [118, 130, 131].

Glucocorticoids are commonly used in the treatment of IBD [132]. Xu et al. observed the penetration of FITC-Dextran 4 (FD4) markers in the lumen of intestinal organoids using coaggregation microscopy [133]. It was found that exposure of the organoid to the glucocorticoid prednisolone significantly reduced intraluminal FD4 infiltration, and decreased inflammatory cytokine expression in the culture medium, indicating that glucocorticoids are well suited to suppress inflammation and reduce intestinal epithelial permeability in IBD treatment [133]. One study added azathioprine and 5-aminosalicylic acid to TNF-α-treated intestinal organoid medium, respectively [134]. The results revealed that TNF-α-treated intestinal organoids showed internalization and abnormal disruption of E-calmodulin and reduced bridging granule core protein-2 levels. The addition of 5-aminosalicylic acid or azathioprine treatment restored E-calmodulin levels on cell membranes. Addition of 5-aminosalicylic acid restored bridging granule core protein-2 levels [134]. Studies have shown that azathioprine and 5-aminosalicylic acid can repair the integrity of the intestinal barrier [134, 135]. Infliximab is an anti-tumor necrosis factor agent that can be used in the treatment of IBD [136]. One study examined the effect of infliximab on IEC using intestinal organoids from UC patients. The results found that concomitant treatment of UC patient’s organoids with infliximab and TNF-α had no significant effect on their viability or morphology, but resulted in a significant decrease in ubiquitin D (UBD) mRNA expression [136, 137]. UBD is a ubiquitin-like modifier involved in protein degradation and is upregulated in inflamed intestinal tissues, suggesting an anti-inflammatory effect of infliximab [136]. Lloyd et al. used healthy human-derived colonic organoids organs incorporating the macrolide clarithromycin and found that clarithromycin exerted anti-inflammatory effects in the intestinal epithelium [138]. This suggests that in addition to its antibacterial effects, clarithromycin has the potential to inhibit the inflammatory response of the intestinal epithelium [138]. Table 2 Novel molecular targets and therapy approach identified using intestinal organoids.

**Table 2:** Novel molecular targets and therapy approach identified using intestinal organoids.

molecular targets	effect	Drug	Species	Ref.
Molecular target identified by treatment of intestinal organoids with current therapies for IBD				
E-cadherin	Re-distribution of protein on intestinal surface restored correct permeability	5-aminosalycilic acid, azathioprine	Mouse (IL-10^−/−^)	[134]
Desmoglein-2	Restored physiological desmoglein-2 expression levels	5-aminosalycilic acid	Mouse (IL-10^−/−^)	[134]
UBD	Restored physiological UBD expression levels	Infliximab	Human (UC patients)	[136]
CLDN-2	Restored physiological CLDN-2 expression levels	Prednisolone, tofacitinib	Human (CD and CRC patients)	[133]
ZO-1	Re-distribution of protein on intestinal surface restored correct	Permeability, Tofacitinib	Human (CRC patients)	[185]
Novel potential molecular targets identified using intestinal organoids				
LRH-1	Improved resistance to pro-inflammatory mediators and induced mucosal healing	**—**	Humanized mouse (Lrh-1^−/−^) LRH-1(^+/+^) and Human	[186]
PXR	Reduced NF-kB activity	**—**	Human (IBD patients)	[187]
IL-22-pSTAT3 SP	Restored tissue damage and intestinal homeostasis	**—**	Mouse (ATF3^−/−^)	[188]
TGF-β SP	Arrested inflammatory signals	**—**	Mouse	[189]
SIRT2	Regulated Wnt/β-catenin SP	**—**	Mouse (Sirt2^−/−^)	[190]
Potential therapeutic approaches for IBD treatment identified using intestinal organoids				
Sex hormones	Decreased expression of ER stress markers	**—**	Human (UC female patients)	[191]
Naltrexone	Reduced ER stress levels, increased the expression of endogenous encephalins and endorphins	**—**	Human (IBD patients)	[192]
*Bacillus subtilis* (RZ001)	Promoted intestinal mucosa repair	**—**	Mouse	[193]
Bacterial indoleacrylic acid	Promoted anti-inflammatory cytokines secretion while inducing goblet cells differentiation	**—**	Mouse	[194]
Hyaluronan 35 kDa	Promoted epithelial wound healing	**—**	Mouse	[195]

*ATF3* Activating transcription factor 3, *CD* Crohn’s disease, *CLDN-2* Claudin-2, *CRC* Colorectal cancer, *ER* Endoplasmic reticulum, *IBD* Inflammatory bowel disease, *IL* Interleukin, *IL-10* Interleukin-10, *LRH-1* Liver receptor homolog 1, *NF-kB* nuclear factor kappa-B, *PXR* Pregnane X receptor, *SIRT2* Human sirtuin protein 2, *SP* Signaling pathway, *STAT* Signal transducer and activator of transcription, *TGF-β*Transforming growth factor-β, *UBD* Ubiquitin D, *UC* Ulcerative colitis, *ZO-1* Zonula occludens.

### Repair of intestinal epithelial injury

**Fig. 3:**
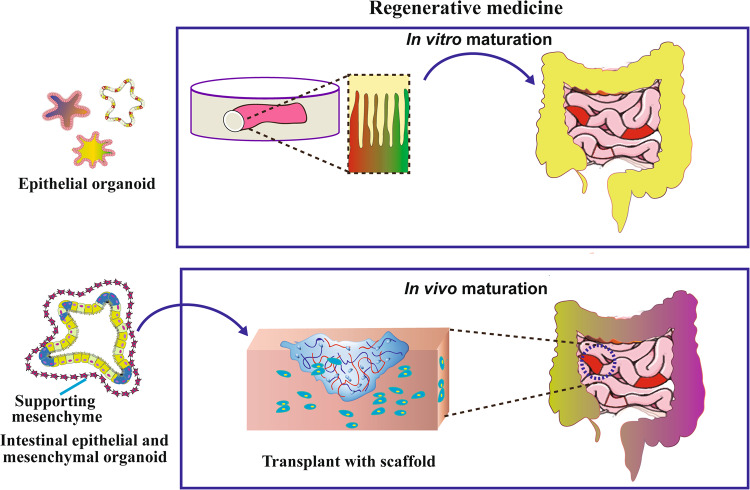
Transplantation of intestinal organoids for the regenerative medicine of ulcerative colitis tissues. Combining organoids with 3D scaffold material can induce the maturation of intestinal organs into functional intestines by implantation into the host. Also in vitro maturation of intestinal organs could repair the original intestinal structure and function.

IBD presents as chronic inflammatory colonic injury, most notably colonic epithelial injury. In non-surgical resection therapy, there is a lack of effective treatment to repair chronic colonic injury and maintain the integrity of the intestinal epithelial barrier [2]. SCs therapy has been shown to be effective in the treatment of IBD, maintaining prolonged remission, repairing intestinal damage, and achieving mucosal healing (MH) [139, 140]. Intestinal organoid transplantation has significant advantages over stem cell transplantation [141]. Colonic organoids are derived from colonic SCs, which have colonic epithelial-like structural features and physiological functions, and retain genetic characteristics [11]. Transplantation into the body is more likely to integrate into the damaged colonic epithelial tissue and achieve the therapeutic goal of MH by regenerating and renewing intestinal epithelial cells and repairing colonic epithelial damage [140, 142, 143]. Figure 3 shows regenerative medicine for intestinal diseases. The development of direct repair of injured epithelial cells or partial replacement of abnormal intestinal epithelial cells with normal epithelial cells would be promising new therapeutic approaches for IBD. Organoid cells can also be transplanted in vivo, which provides a pre-clinical tool for regenerative medicine. For example, one study described in detail how epithelial organoids were transplanted into the colon of a mouse model of inflammatory enteritis. In this experiment, they injected the organoids into the luminal space at the anus. The injected organoids then attach to the injured area and reconstruct the donor-derived epithelium. This method has been successfully applied to epithelial cell-derived organoid tissue from adult colon and small intestine epithelium as well as fetal small intestine [144]. It has been shown that intestinal organoids are implanted through the anus into the dextran sodium sulfate(DSS)-induced colitis model, the clinical activity score can be significantly improved, and the donor cells can be accurately targeted to localize in the colitis-induced ulcer surface, and begin to reconstruct and repair the crypt structure [145–147]. In 2012, Yui et al. reported that in vitro cultured intestinal organoids were able to repair colitis through anal enucleation in mice, and the donor cells were able to target the colonic ulcer surface and start to reconstruct and repair the crypt structure [142]. In 2022, the same mouse-derived intestinal organoids were injected anally into UC mice by Tokyo Medical and Dental University (TMDU), Japan, to repair colitis, and these cultured organoids could precisely reach the location in the damaged intestinal epithelium and repair the damaged intestinal epithelium [148]. Subsequently, the TMDU research team announced the first successful transplantation of "organoids" into the intestinal mucosa of a patient with refractory UC [149]. The "organoid" is derived from the patient’s normal intestinal mucosa (including ISCs), and cultured in vitro in 3D to form 0.1–0.2 mm diameter "organoids", which are then endoscopically transplanted to the colon lesion site [148, 149]. Periodic endoscopic observation of mucosal repair and improvement of colonic lesions was performed. In addition, ISCs secrete a large number of extracellular vesicles in the culture medium during the formation of intestinal organoids [150–152]. The extracellular vesicles extracted from the culture medium have regulatory repair functions similar to those of mother cells [153–155]. It can be used as cell-free therapy and applied in IBD treatment, which can achieve the effects of repairing intestinal epithelium, promoting mucosal healing, and regulating immune dysfunction [152, 155, 156].

### Gene therapy

Organoid culture technology can be combined with gene editing technology. The gene editing technology represented by clustered regularly interspaced short palindromic repeats-associated protein 9 (CRISPR/Cas9) is becoming mature [157–160]. Gene editing of SCs or organoids using CRISPR/Cas9 for genetic modification of intestinal organoids [157, 161]. Genetic correction is performed in the case of genetic defects to achieve improvement of genetic susceptibility factors for IBD, prevention of carcinogenesis, or inhibition of tumor growth [94, 162, 163]. Alternatively, gene expression in intestinal organoids can be altered by DNA or RNA transfection, lentiviral infection, and other methods [164, 165]. Clevers et al. removed a small number of ISCs from patients with Cystic fibrosis (CF), modified the genes of these SCs using CRISPR/Cas9 technology, inserted normal cystic fibrosis transmembrane conductance regulator (CFTR) genes, and made them develop into organoid. These organoids, if reintroduced into the source patient, may partially cure CF disease [166].

## Limitations of intestinal organoids technology

The organoid has more than 10 years of development and fruitful research results, but there are still certain limitations [13, 29, 167].

### Potential hazards

In the process of 3D culture and gene editing, the differentiation of SCs and genetic modification needs to be precisely regulated, and there is a possibility of abnormal differentiation and gene mutation of SCs, and the chance of tumor formation increases. Allogeneic organ transplantation requires consideration of immunogenicity, which poses certain safety risks [13, 167]. Regarding culture systems and culture additives, it is important to guard against microbial contamination. It is important to consider the toxic components contained in synthetic materials and the immunogenicity of natural biological materials [67].

### Tissue differences

Current organoid culture systems do not completely mimic the complex growth environment of the in vivo intestine. In comparison with the in vivo intestine, intestinal organoids also have certain tissue differences and functional defects. The intestinal organoid is dominated by intestinal epithelium and lacks connective, muscular, and neural tissues. There are no blood vessels, lymphatic vessels, and neurogenesis, and a lack of immune cells and smooth muscle cells [54].

### Defective culture system

Materials and techniques are the basis for establishing culture systems. With the development and improvement of materials, diverse culture systems can be established, but all have different degrees of defects. It is still technically difficult to completely simulate the complex environment in vivo and monitor the culture process in real time, and there is still a long way to go before the integrated culture and commercial application of intestinal organoids [47, 61, 70].

### Organoid heterogeneity

Both cell source and culture conditions affect the structure and function of organoids. At present, the sources of SCs are diverse, the cultural techniques are not uniform, and the research results are obviously individual. There are certain obstacles to the sharing of results and technology diffusion [43].

## Summary

Currently, organoids have a wide range of applications in organ development, precision medicine, regenerative medicine, drug screening, gene editing, and disease modeling. SCs-derived intestinal organoid is an ideal intestinal epithelial physiological model and intestinal disease model, which has obvious advantages in individualized disease modeling and precision medicine. As a model of IBD disease, it can explore the pathogenesis of IBD, test the therapeutic effect of IBD, and find new therapeutic targets again. Intestinal organoids transplanted into IBD patients can repair intestinal epithelial damage. After gene therapy, intestinal organoid transplants can also improve IBD genetic susceptibility and improve gene expression. Intestinal organoid-derived extracellular vesicles have also demonstrated a role in repairing intestinal damage and regulating immune disorders.

However, the limitations of the intestinal organoid limit its clinical translation, and continuous research is still needed to break through its limitations. First of all, we need to expand the scope of research, conduct more extensive model studies, explore new therapeutic approaches, and prove the effectiveness, safety, and feasibility of organoid applications. Within the scope of ethics, we should optimize organoid transplantation and gene therapy technology, and conduct more animal or clinical trials to promote the clinical translation of organoids. Secondly, we need to optimize the culture system. Through material and technical improvements, we can better simulate the in vivo intestinal physicochemical and microbial environment, homogenize culture, and narrow the differences between organoid and in vivo organs. To simulate in vivo cellular interactions and establish a multicellular co-culture system in vitro. Table 3 Overview of the currently established organoid–cell co-culture systems. With the breakthrough of technical and ethical limitations, the clinical translation of intestinal organoids can be widely carried out. The future of intestinal organoids can bring a bright future to the research and treatment of IBD.

**Table 3:** Overview of the currently established organoid–cell co-culture systems.

Intestinal cells	Co-culture	Device material	Technique	Major observations	Ref.
Caco-2	*L. rhamnosus*	PDMS, type I collagen, and Matrigel mix	Soft lithography	Fluid flow accelerated intestinal epithelial differentiation and organization into villi-like structures, mechanical stimulation enhanced specific differentiation features, sustained long-term co-culture with commensal bacteria	[196]
Human duodenal organoids (pediatric donors)	HIMECs	PDMS, type I collagen, and Matrigel mix	Soft lithography	Transcriptome of the intestinal tissue-on-chip more closely resembled that of the duodenum in vivo than the initial organoid culture from which it was derived, co-culture with endothelial cells accelerated the formation of the epithelial monolayer	[184]
Human duodenal organoids (adult donors)	HIMECs	PDMS, type IV collagen, and Matrigel mix	Soft lithography	Showed culture system suitability for studying intestinal metabolism and drug transport	[197]
Human jejunal organoids (adult donors)	HUVECs	PDMS, type IV collagen	Soft lithography	Shear stress generated by luminal and basolateral flow produced a model of continuous intestinal differentiation, no villi-like structures observed with stem cell expansion media on the luminal side	[198]
Mouse colon tissue explant	Intestinal submucosal and muscular layers, microbiota	Cyclin olefin polymer and polyurethane no ECM	Injection molding	Dual-flow microfluidics allowed for the culture of full thickness explants over 3 days, recapitulated the in vivo oxygen gradient across the epithelial layer	[199]
Mouse proximal small intestine organoids	Cryptosporidium parvum	PDMS, Type I collagen, and Matrigel mix-coated 3D scaffold	Soft lithography and laser ablation	Established a long-lived and tube-shaped intestinal epithelial culture system by using crypt-like microcavities under flow, induced topography-guided self-organization of a functional epithelium with crypt- and villus-like domains similar to that observed in vivo, the culture system showed self-regeneration capacity and response to bacterial infection	[200]
Caco-2	*L. rhamnosus* GG and *Bacteroides caccae*	Polycarbonate, type I collagen; porcine gastric mucin	Computer-controlled milling, laser cutting, and bolting	Engineered a modular architecture consisting of 3 microchambers to facilitate human and microbial cell interface, allowed measuring individual transcriptional responses in different infectious contexts and real-time monitoring of oxygen concentrations	[201]
Caco-2	Human gut microbiota, *E. coli*, human PBMCs, human microvascular endothelial cells, and human lymphatic microvascular endothelial cells	PDMS, type I collagen, and Matrigel mix	Soft lithography	Established a stable long-term co-culture system of commensal and pathogenic microbes with intestinal epithelial cells, lack of mechanical stimulation induced bacterial overgrowth, similar to what is observed in IBD patients, emulated intestinal infection and inflammatory responses	[180]
Caco-2	Human gut microbiota, *E. coli*, PBMCs	PDMS, type I collagen, and Matrigel mix	Soft lithography	Re-created a dextran sodium sulfate–induced epithelial inflammatory response, described intestinal barrier dysfunction as a critical trigger of inflammation onset in the gut	[202]
Caco-2	*S. flexneri*	PDMS, type I collagen, and Matrigel mix	Soft lithography	Enabled the replication of Shigella infection hallmarks, Shigella invaded directly via the luminal side of the epithelium composed solely of enterocytes, 3D crypt-like structures provided a safe harbor for bacteria against luminal washout	[203]
Human colon organoids (pediatric and adult donors)	HIMECs, EHEC	PDMS, type I collagen, and Matrigel mix	Soft lithography	Observed that infectious activity of EHEC is promoted by human gut microbiome metabolites, when compared with those derived from mouse, recapitulated the proinflammatory and anti-inflammatory cytokine profiles induced by EHEC infection	[204]
Caco-2	*Bifidobacterium adolescentis* and *Eubacterium hallii*	PDMS, type I collagen and Matrigel mix	Soft lithography	Simulated a steady-state vertical oxygen gradient, the transepithelial anoxic interface allowed co-culture with obligate anaerobes	[205]
Caco-2 and human ileal organoids (pediatric donors)	*Bacteroides fragilis*, human gut microbiota, HIMECs	PDMS, type I collagen, and Matrigel mix	Soft lithography	Established an oxygen gradient compatible with co-culture of a complex community of anaerobic commensal microorganisms	[183]
Caco-2	HUVECs, PBMCs, mucosal macrophages, dendritic cells, *L. rhamnosus*, *Candida albicans*	Polystyrol, PET	Injection molding	Characterized immunologic responses to luminal lipopolysaccharide and endotoxemia, addressed the role of probiotics in protecting from opportunistic infections	[206]
Caco-2	Neutrophils, monocytic THP1 cells	Glass, polystyrene, and proprietary polymers Membrane-free (Phase Guide) type I collagen	—	Simulated acute intestinal inflammatory responses by enabling neutrophil recruitment to the parenchymal compartment	[38]
Human colon organoids (pediatric and adult donors)	PBMCs	Glass, polystyrene, and proprietary polymers type I collagen	—	Studied IBD-associated inflammatory responses	[207]
Human ileal organoids (pediatric donors)	Endothelial colony forming cell derived endothelial cells, intestinal subepithelial myofibroblasts	PDMS, Polycarbonate type IV collagen	Soft lithography	Characterized ISEMF-induced angiogenesis and its physiological parameters, evaluated the effect of perfused vasculature on intestinal epithelial cell culture	[208]
Mouse small intestine	Lamina propria lymphocytes and ILC3s	recombinant mouse IL-2, IL-7, IL-15 and IL-23	—	IL-22-dependent stem cell proliferation	[123]
Mouse small intestine	Pre-stimulated CD4+ splenocytes	Organoid medium	—	Intestinal stem cell differentiation	[119]
Human fetal intestine	Pre-stimulated fetal lamina propria T cells	p38 MAPK inhibitor, recombinant human IL-2	—	Organoid outgrowth	[209]
Mouse small intestine	αβ and γδ IELs	recombinant mouse IL-2, IL-7, IL-15	—	IEL survival, proliferation, and incorporation in the epithelium	[210]
Mouse small intestine	OT-I CD8+ splenocytes	EGF, noggin, R-spondin1 or R-spondin3	—	T cell proliferation and IEL phenotype	[211]
Mouse Lgr5(+) ISCs at single-cell level	OT-II splenocytes (naive)	Organoid medium	—	T cell proliferation	[119]

*CAR* chimeric antigen receptor, *CDK* cyclin-dependent kinase, *CRC* colorectal cancer, *DC* dendritic cell, *DKK1* dickkopf Wnt signaling pathway inhibitor 1, *ECM* extracellular matrix, *EGF* epidermal growth factor, *EHEC* enterohemorrhagic E coli, *FZD9* frizzled class receptor 9, *HIMEC* human intestinal microvascular endothelial cell, *HUVEC* human umbilical vein endothelial cell, *IEL* intraepithelial lymphocyte, *ILC3* group 3 innate lymphoid cell, *ISEMF* intestinal subepithelial myofibroblast, *MAPK* mitogen-activated protein kinase, *MDOTS* mouse-derived organotypic tumor spheroid, *MUC2* mucin 2, *NSCLC* non-small-cell lung cancer, *PD1* programmed cell death 1, *PDMS* polydimethylsiloxane, *PDOTS* patient-derived organotypic tumor spheroid, *PET* polyethylene terephthalate, *RSV* respiratory syncytial virus, *TCR* T cell receptor, *THP1* Tohoku Hospital Pediatrics-1 cell line, *WNT3A* WNT family member 3A.
